# Genome-wide association study reveals the genetic basis of yield- and quality-related traits in wheat

**DOI:** 10.1186/s12870-021-02925-7

**Published:** 2021-03-19

**Authors:** Le Gao, Chengsheng Meng, Tengfei Yi, Ke Xu, Huiwen Cao, Shuhua Zhang, Xueju Yang, Yong Zhao

**Affiliations:** State Key Laboratory of North China Crop Improvement and Regulation, Hebei Agricultural University, Baoding, 071000 Hebei China

**Keywords:** GWAS, Quality, Single nucleotide polymorphism, Wheat, Yield

## Abstract

**Background:**

Identifying the loci and dissecting the genetic architecture underlying wheat yield- and quality-related traits are essential for wheat breeding. A genome-wide association study was conducted using a high-density 90 K SNP array to analyze the yield- and quality-related traits of 543 bread wheat varieties.

**Results:**

A total of 11,140 polymorphic SNPs were distributed on 21 chromosomes, including 270 significant SNPs associated with 25 yield- and quality-related traits. Additionally, 638 putative candidate genes were detected near the significant SNPs based on BLUP data, including three (*TraesCS7A01G482000*, *TraesCS4B01G343700*, and *TraesCS6B01G295400*) related to spikelet number per spike, diameter of the first internode, and grain volume. The three candidate genes were further analyzed using stage- and tissue- specific gene expression data derived from an RNA-seq analysis. These genes are promising candidates for enhancing yield- and quality-related traits in wheat.

**Conclusions:**

The results of this study provide a new insight to understand the genetic basis of wheat yield and quality. Furthermore, the markers detected in this study may be applicable for marker-assisted selection in wheat breeding programs.

**Supplementary Information:**

The online version contains supplementary material available at 10.1186/s12870-021-02925-7.

## Background

Bread wheat (*Triticum aestivum* L.), which is a widely cultivated cereal crop that is highly adaptable, provides approximately 21% of the total calories and 23% of protein in the human diet (www.fao.org/faostat/en). As a staple food for about 35–40% of the global population, wheat is a good source of nutrients and has unique gluten properties, making it useful for producing diverse food products [1]. The increasing global population and improvements in the standard of living for many people worldwide have forced breeders to continually aim to produce new high-quality and high-yielding wheat varieties [2].

Yield and quality are complex traits. Additionally, the limited genetic diversity of bread wheat has resulted in breeding bottlenecks, and the application of traditional breeding methods has led to gradual increases in wheat yield and quality [3]. Genome sequencing and high-throughput chip-based genotyping platforms are critical for clarifying the mechanisms regulating the wheat yield potential and quality as well as for enhancing breeding methods [4]. Several SNP arrays (e.g., 9 K, 35 K, 90 K, 660 K, and 820 K) have recently been developed. They have been used to analyze bi-parental populations and identify loci (QTLs) controlling yield- and quality-related traits [5–9]. However, traditional QTL mapping methods are usually based on specific characteristics of parental populations, and are time-consuming and laborious [10].

GWAS are common in breeding programs because they are more efficient and require less effort in analyzing complex traits under various environmental conditions than other research methods [11]. Specifically, GWASs have been useful for detecting yield-associated loci in wheat, including plant height (PH), kernel number per spike (KNPS) and thousand grain weight (TGW) [12, 13]. However, because of the need for many seeds and the substantial time required to assess some quality traits, there have been relatively few GWASs regarding wheat quality traits such as wet gluten content (WGC) and grain protein content (GPC) [14, 15]. Moreover, there are few reports describing a GWAS conducted to investigate lodging resistance, which is an important factor influencing wheat yield and quality.

For a GWAS, the size and diversity of the panel are important because a small panel and large linkage disequilibrium (LD) blocks may lead to the identification of false positive associations [16]. Regarding wheat, only a few GWAS for yield and quality traits have involved large natural populations and SNP chips. Furthermore, wheat has been cultivated in China for more than 4000 years and has now been cultivated in 10 major agro-ecological zones [17]. Due to the long evolutionary period, Chinese wheat germplasms have been artificially selected in different regions and have regional genetic characteristics [18, 19]. Accordingly, the objectives and requirements for improving wheat varieties differ considerably among these regions. Thus, we performed a GWAS of wheat yield and quality involving 543 representative bread wheat cultivars, including 531 Chinese wheat cultivars from 10 provinces, and a wheat 90 K SNP array following phenotypic analyses in six environments.

The aim of this study was to identify the stable loci and candidate genes significantly associated with wheat yield and quality. The results described herein may be useful for revealing the genetic basis of yield and quality. The corresponding SNP markers that were identified may ultimately facilitate the breeding of new high-quality and high-yielding wheat varieties.

## Results

### Phenotypic variation and correlation analysis

The phenotypic data for the 543 wheat lines characterized regarding growth- and development-related traits, yield-related traits, and quality-related traits in six environments are listed in Table S1. The phenotypic variations among genotypes were determined based on the heritability, range, mean, standard deviation, and the coefficient of variation. There were obvious variations for all traits, especially the coefficient of variation for thrust (TH) (49.28%) in the E5 environment. Table S2 provides the estimated correlation coefficients for this combined analysis. The broad-sense heritability (h^2^) for most traits was approximately 0.80, with the highest and lowest heritabilities detected for PH (0.92) and PET (0.70). Accordingly, most traits were stable and largely determined by genetic factors. The correlation coefficient was highest (0.970) between wet gluten content (WGC) and grain protein content (GPC), but was also relatively high between TGW and GPR (0.919), wet gluten content (WGC) and flour content (FC) (0.842).

### Genome-wide association study

The 90 K wheat iSelect SNP array with 81,587 SNPs was used for genotyping. After a quality control step, 11,140 SNP markers remained for the association mapping [20]. A total of 270 significant SNP loci associated with yield and quality traits were identified (Table S3, Fig. S1). These SNPs were located on 21 chromosomes and accounted for 1.27–8.47% of the phenotypic variation. Moreover, 94, 139, and 37 SNPs were in the A, B, and D subgenomes, respectively (Table S3). Of these SNPs, 42 pleiotropic loci associated with two or more traits were detected on chromosomes 1B, 2A, 2B, 2D, 3A, 3B, 3D, 4B, 4D, 5B, 5D, 6B, 6D, 7A, and 7B based on the common loci (Table 1).

**Table 1:** SNP sites detected in two or more traits

SNP	Trait	Environment	Chromosome	Position	-log_10_p	R^2^(%)
RAC875_rep_c111906_144	FLL/FLA	E2	2A	27,967,266	4.45–4.95	2.84–3.23
Tdurum_contig19022_1524	FLL/FLA	E2	7B	530,376,060	4.13–4.24	2.61–2.68
wsnp_CAP11_c2435_1256981	FLL/FLA/FLW	E2E5	7A	655,447,518	5.76–7.74	3.79–5.32
GENE-0035_150	FLL/FLA/FLW/MTN	E2E5	1B	465,098,532	4.22–6.73	2.65–4.55
GENE-0993_47	FLL/FLA/FLW/MTN	E2E5	2B	55,691,934	4.68–7.52	2.99–5.16
Tdurum_contig73039_241	FLL/FLA/FLW/MTN	E2E5	3B	527,296,306	5.03–7.47	3.25–5.12
Tdurum_contig4974_355	FLL/FLA/FLW/MTN	E2E5	4B	95,708,069	4.71–7.29	3.01–4.98
Ku_c7989_781	FLL/FLA/FLW/MTN	E2E5	5B	253,378,428	4.28–5.77	2.7–3.82
D_contig73483_655	FLL/FLA/FLW/MTN	E2E5	5D	100,546,173	4.7–7.93	3.01–5.47
GENE-3803_329	FLL/FLA/FLW/MTN	E2E5	6B	556,487,785	4.14–5.61	2.59–4.37
BobWhite_c36864_159	FLL/FLA/FLW/MTN	E2E5	7B	131,745,465	4.19–6.86	2.63–4.85
Tdurum_contig41918_2469	FLL/FLA/FLW/MTN	E2E5	7B	628,465,395	4.74–7.57	3.03–5.20
Tdurum_contig42029_1151	FLL/FLA/FLW/MTN	E2E5	7B	660,237,019	4.78–7.64	3.07–5.25
BS00099128_51	FLW/MTN	E2E5	2D	470,217,019	4.27–4.73	2.91–3.23
Tdurum_contig17320_458	FLW/MTN	E2E5	3A	170,784,653	4.12–6.46	2.80–4.61
Excalibur_c43604_751	FLW/MTN	E2E5	3B	807,286,509	4.16–6.97	2.83–5.02
Tdurum_contig100205_499	FLW/MTN	E2E5	4B	78,818,313	4.38–6.7	3–4.8
wsnp_Ex_c402_791233	FLA/FLW	E2E5	1B	548,623,447	5.51–6.37	3.87–4.34
Excalibur_c89155_115	FLA/FLW	E5	6B	619,483,986	4.1–4.36	2.78–2.8
GENE-1343_878	FLA/FLW/MTN	E2E5	2D	13,440,948	5.10–6.4	3.47–4.3
D_F1BEJMU01DOWJ3_176	FLA/FLW/MTN	E2E5	2D	138,694,869	5.49–6.38	3.66–4.46
GENE-0826_51	FLA/FLW/MTN	E2E5	3A	32,151,249	4.94–6.07	3.23–4.09
Tdurum_contig16643_466	FLA/FLW/MTN	E2E5	3A	549,267,983	4.49–5.85	2.89–4.12
D_GBF1XID01CVZMX_132	FLA/FLW/MTN	E2E5	5D	483,515,041	4.89–6.97	3.2–5.02
IACX1201	FLA/FLW/MTN	E2E5	6B	219,580,396	4.06–5.96	2.65–4.2
D_contig38762_578	FLA/FLW/MTN	E2E5	6D	67,378,176	5.39–6.66	3.75–4.49
RAC875_c48208_304	FLA/FLW/MTN	E2E5	7B	3,505,857	5.28–6.17	3.49–4.13
GENE-4534_455	FLA/FLW/MTN	E2E5	7B	152,051,848	5.33–6.43	3.52–4.32
Tdurum_contig42179_1562	PET/MTN	E2E5	3A	266,433,409	4.71–7.27	3.19–5.26
D_contig26931_415	PET/MTN	E2E5	5D	283,010,539	4.12–7.34	2.74–5.32
wsnp_Ex_c14654_22713386	PET/MTN	E2E5	7A	11,098,761	4.53–7.07	3.05–5.1
BS00044895_51	PET/MTN	E2E5	7A	211,733,339	4.35–7.05	2.91–5.08
Tdurum_contig19022_1524	PET/MTN	E2E5	7B	530,376,060	4.6–5.51	3.13–3.85
Kukri_c56333_138	MTN/FC	BLUP	1B	670,176,213	4.50–4.75	1.80–2.77
BobWhite_c19617_154	GFR/TGW	BLUP	2B	209,099,527	4.89–4.91	3.46–3.59
Kukri_c322_1394	FD/SD	E1/BLUP	4B	520,238,759	4.64–4.8	3.07–4.07
Tdurum_contig48366_1324	FD/SD	E1E2/BLUP	4B	637,387,355	4.13–4.88	2.62–5.02
Tdurum_contig50783_67	FD/SD	BLUP	4B	637,387,809	4.07–4.24	2.57–3.11
RAC875_rep_c105718_585	FIL/SIL/PH	E3E4E5E6/BLUP	4D	25,989,112	4.09–7.89	2.03–7.56
BS00044895_51	FIL/SIL/TH	E1E2	7A	211,733,339	4.4–6.47	3.78–5.97
BS00022854_51	SIL/TH	E1E5	5B	614,983,507	4.29–6.13	2.7–5.62
Ex_c52589_795	GPC/WGC	E1	3D	531,375,739	4.36–4.58	2.48–2.53

### Growth and development-related traits

A total of 28 significant SNP loci for the flag leaf length (FLL) were detected on chromosomes 1B, 1D, 2A, 2B, 2D, 3A, 3B, 4B, 5A, 5B, 5D, 6B, 6D, 7A, and 7B, accounting for 2.44–4.12% of the phenotypic variation. Regarding the flag leaf width (FLW), 33 significant SNP loci were detected on 13 chromosomes (1B, 2B, 2D, 3A, 3B, 4A, 4B, 5B, 5D, 6B, 6D, 7A, and 7B) and explained about 2.52–6.92% of the phenotypic variation. For the flag leaf area (FLA), the 40 significant SNP loci identified across six environments were detected on 16 chromosomes (1B, 2A, 2B, 2D, 3A, 3B, 3D, 4A, 4B, 4D, 5B, 5D, 6B, 6D, 7A, and 7B) and explained about 2.58–6.37% of the phenotypic variation. For the flag leaf angle (FA), 13 significant SNP loci were detected on nine chromosomes (1B, 2B, 3A, 3B, 4B, 4D, 5A, 6A, and 6D), accounting for about 2.06–3.35% of the phenotypic variation. Of the 68 SNPs identified for the flag leaf-associated traits, 11 pleiotropic loci were associated with three traits.

For the maximum tiller number (MTN), 37 significant SNP loci were detected on 14 chromosomes (1A, 1B, 2B, 2D, 3A, 3B, 4B, 4D, 5B, 5D, 6B, 6D, 7A, and 7B) and explained about 1.80–5.32% of the phenotypic variation. Five significant SNP loci for the heading date (HD) were distributed on chromosomes 2A and 5A, accounting for 2.73–5.94% of the phenotypic variation. Seven significant SNP loci for the mature period (MP) were detected on chromosomes 2B, 3B, 6A, and 7B and accounted for 2.70–3.75% of the phenotypic variation. The seven significant SNP loci for the grain-filling period (GFP) were detected on chromosomes 2A, 2B, 2D, and 7B and accounted for 1.37–2.99% of the phenotypic variation. Nine significant SNP loci for the grain-filling rate (GFR) were detected on chromosomes 1A, 1B, 2A, 2B, 4A, 4B, and 5B, accounting for 2.39–3.59% of the phenotypic variation. Eight significant SNP loci for TGW were detected on chromosomes 1B, 2B, 3A, 3B, 4B, and 6D, explaining 2.29–3.59% of the phenotypic variation.

For PH, 20 significant SNP loci were detected on 10 chromosomes (1A, 1B, 2B, 3A, 3B, 4A, 4B, 4D, 5A, and 6B) and explained about 1.96–4.42% of the phenotypic variation. For the diameter of the first internode (FD), 16 significant SNP loci were detected on nine chromosomes (1A, 1B, 2B, 3D, 4A, 4B, 5B, 6A, and 7A) and explained about 1.53–4.85% of the phenotypic variation. Regarding the length of the first internode (FIL), 17 significant SNP loci were detected on eight chromosomes (1A, 1B, 2A, 3A, 3B, 4D, 6B, and 7A) and explained about 2.13–5.76% of the phenotypic variation. For the diameter of the second internode (SD), the 18 significant SNP loci identified across six environments were detected on eight chromosomes (2A, 2B, 2D, 3B, 4B, 5B, 6B, and 7B), explaining about 2.15–5.02% of the phenotypic variation. For the length of the second internode (SIL), 15 significant SNP loci were detected on seven chromosomes (1A, 1D, 4B, 4D, 5B, 7A, and 7D) and explained about 2.57–5.74% of the phenotypic variation. For TH, 45 significant SNP loci were detected on 15 chromosomes (1B, 2B, 2D, 3A, 3B, 4A, 4B, 4D, 5B, 5D, 6A, 6B, 6D, 7A, and 7B), accounting for approximately 2.5–7.13% of the phenotypic variation. Among them, it is noteworthy that three loci on chromosome 4B are associated with FD and SD.

### Yield-related traits

For spike length (SL), nine significant SNP loci were distributed on seven chromosomes (1B, 3A, 3D, 4B, 6D, 7A, and 7B) and explained about 2.42–4.33% of the phenotypic variation. Regarding the spikelet number per spike (SNS), 14 significant SNP loci were detected on six chromosomes (2A, 2B, 2D, 4B, 4D, and 7A), explaining about 1.27–4.02% of the phenotypic variation. The nine significant SNP loci for KNPS were detected on chromosomes 1A, 3A, 4A, 4B, and 5D and accounted for 2.58–4.32% of the phenotypic variation. Regarding the percentage of spike-bearing tillers (PET), 11 significant SNP loci were detected on 10 chromosomes (1A, 2A, 3A, 4D, 5B, 5D, 6B, 6D, 7A, and 7B) and explained about 2.74–3.55% of the phenotypic variation. For the spike number per mu (EPM), 17 significant SNP loci were detected on eight chromosomes (1B, 2A, 4B, 4D, 5A, 5B, 7A, and 7B) and explained about 2.33–8.47% of the phenotypic variation.

### Quality-related traits

For the grain volume (GV), 17 significant SNP loci were detected on five chromosomes (1B, 3B, 3D, 6A, and 6B) and explained about 2.52–5.01% of the phenotypic variation. The two significant SNP loci for GPC detected on chromosomes 1A and 3D accounted for 2.35–2.53% of the phenotypic variation. Two significant SNP loci for WGC were detected on chromosomes 3D and 5D and accounted for 2.43–2.90% of the phenotypic variation. Ten significant SNP loci for the flour content (FC) were detected on chromosomes 1B, 4B, 5A, 5B, 6A, and 7B, accounting for 1.80–3.27% of the phenotypic variation.

### Putative candidate gene analysis and expression data

**Fig. 1:**
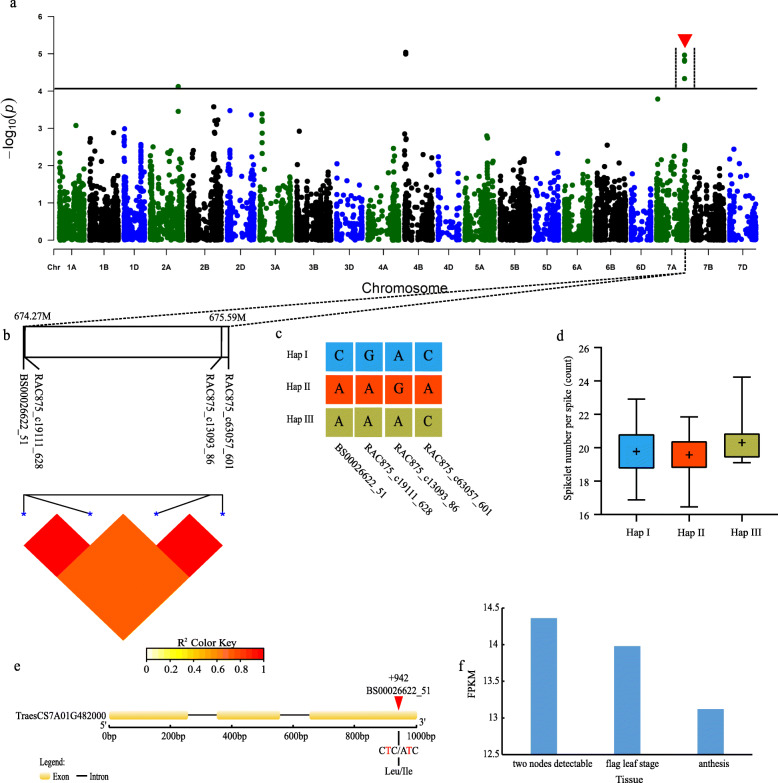
Genome-wide association study results for SNS and an analysis of the peak on chromosome 7A. **a** Manhattan plots for SNS. The horizontal line represents the significance threshold. The arrows indicate the location of the main peaks studied. **b** Genomic locations of four SNP loci and the LD based on paired R^2^ values between SNPs on chromosome 7A. **c** Haplotypes detected in 543 accessions based on the four SNPs. **d** Differences in the SNS among three haplotypes. **e** Structure of the *TraesCS7A01G482000* gene. **f** Transcriptomic analysis of *TraesCS7A01G482000* in the spike during different developmental stages. Gene expression was based on the fragments per kilobase of transcript per million mapped reads (FPKM) value

**Fig. 2:**
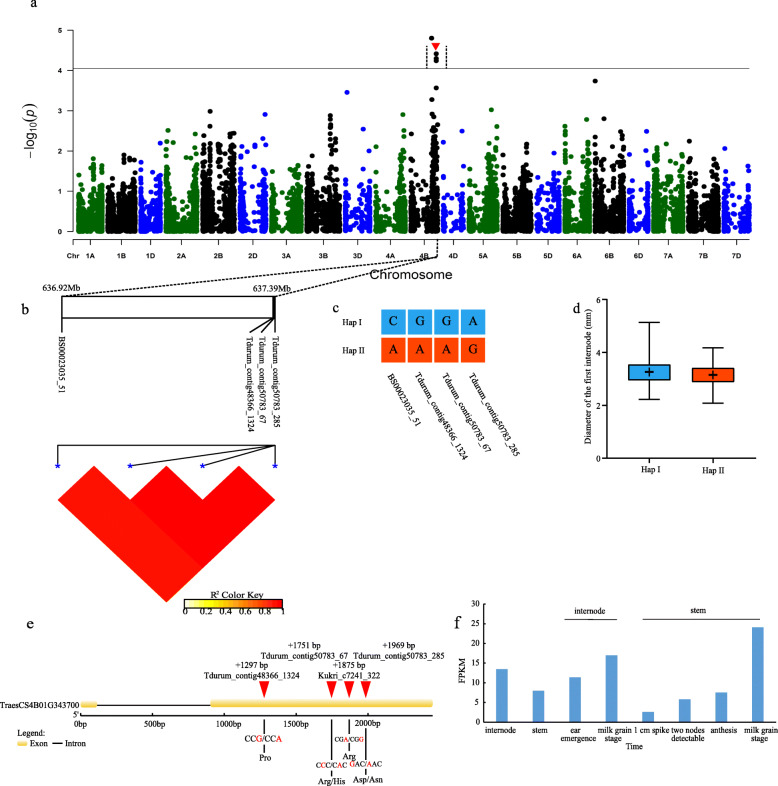
Genome-wide association study results for FD and an analysis of the peak on chromosome 4B. **a** Manhattan plots for FD. The horizontal line represents the significance threshold. The arrows indicate the location of the main peaks studied. **b** Genomic locations of four SNP loci and the LD based on paired R^2^ values between SNPs on chromosome 4B. **c** Haplotypes detected in 543 accessions based on the four SNPs. **d** Differences in the FD between two haplotypes. **e** Structure of the *TraesCS4B01G343700* gene. **f** Transcriptomic analysis of *TraesCS4B01G343700* in different tissues and stages. Gene expression was based on the fragments per kilobase of transcript per million mapped reads (FPKM) value

**Fig. 3:**
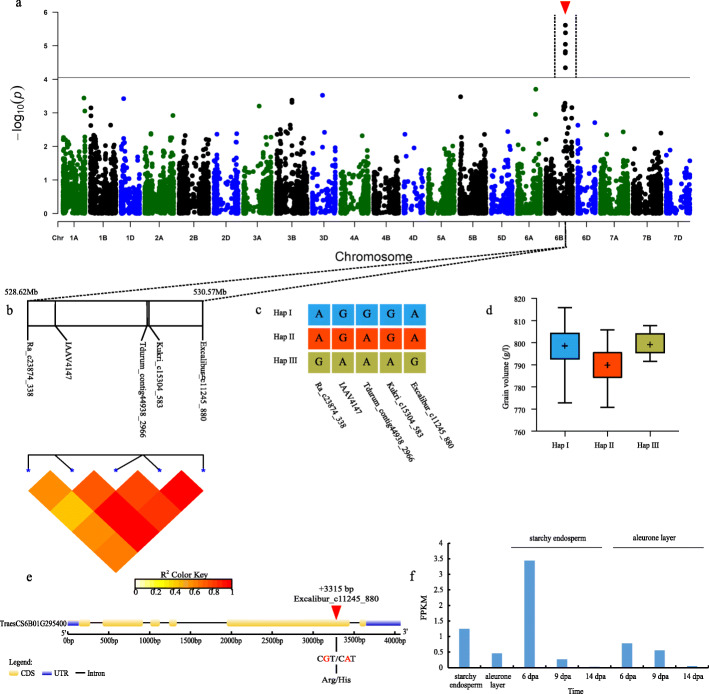
Genome-wide association study results for GV and an analysis of the peak on chromosome 6B. **a** Manhattan plots for GV. The horizontal line represents the significance threshold. The arrows indicate the location of the main peaks studied. **b** Genomic locations of five SNP loci and the LD based on paired R^2^ values between SNPs on chromosome 6B. **c** Haplotypes detected in 543 accessions based on the five SNPs. **d** Differences in the GV among three haplotypes. **e** Structure of the *TraesCS6B01G295400* gene. **f** Transcriptomic analysis of *TraesCS6B01G295400* in different tissues and stages. Gene expression was based on the fragments per kilobase of transcript per million mapped reads (FPKM) value

In our study, the 200-, 380-, and 600-kb sequences flanking the related SNPs in subgenomes A, B, and D, respectively, were identified as potential candidate gene regions. A total of 638 putative candidate genes detected of the significant SNPs flanking-regions based on BLUP data were identified by screening the annotated genes in the recently released genome sequence (IWGSC RefSeq v1.0) (Table S4). We performed subsequent haplotype and expression analysis for the following three critical traits: SNS (Fig. 1), FD (Fig. 2), and GV (Fig. 3).

Regarding the SNPs associated with SNS, an association peak that included four significantly associated SNPs was detected on chromosome 7A (Fig. 1a, b). On the basis of the SNPs on chromosome 7A, three haplotypes were identified, with the mean SNS for haplotype II (19.57 ± 1.14) significantly lower than the corresponding values for haplotypes I (19.78 ± 1.33) and III (20.31 ± 1.43) (Fig. 1c, d). One of the four significant SNPs (BS00026622_51) was located at 942 bp in the third exon of the gene *TraesCS7A01G482000*. This locus was detected in four environments and in the BLUP model. SNP (C/A) at this location in the relevant genomic regions cause amino acids to change from leucine to isoleucine (Fig. 1e). The expression of the *TraesCS7A01G482000* candidate gene when two nodes were detectable was significantly upregulated according to the RNA-seq analysis of the spike (Fig. 1f).

Among the SNPs associated with FD, four similar SNPs were distributed on chromosome 4B and all were detected in the BLUP model and in at least one environment (Fig. 2a). These four SNPs were BS00023035_51, Tdurum_contig48366_1324, Tdurum_contig50783_67, and Tdurum_contig50783_285, and four SNPs were separated 470.67 kb (Fig. 2b). Because of their close genetic relationship and significant correlation, these SNPs were used for the subsequent haplotype analysis, which revealed two distinct haplotypes (I and II). A total of 301 wheat materials were included haplotype I, with an FD of 3.25 ± 0.48 cm, whereas 208 wheat varieties were included haplotype II, with an FD of 3.17 ± 0.45 cm (Fig. 2c, d). The FD of haplotype I was significantly greater than that of haplotype II, implying lodging is less likely for wheat varieties with haplotype I than for varieties with haplotype II. Furthermore, three of the four significant SNPs were detected in the CDS of *TraesCS4B01G343700*. This gene contains two exons and the CDS comprises 2450 bp. The two SNPs located in the second exon resulted in amino acid changes from arginine to histidine and from aspartic acid to asparagine (Fig. 2e). The *TraesCS4B01G343700* expression level increased in the stem and internode during wheat growth and development, peaking in the milk stage. This suggests that this gene helps mediate wheat internode growth and development (Fig. 2f).

The changes in GV were highly correlated with a set of SNPs in a 1.95 Mb genomic region (528.62–530.57 Mb) on chromosome 6B (Fig. 3a, b). These loci were detected in multiallelic and BLUP contexts. Three haplotypes were detected in 407 wheat lines on the basis of genotyping results. More specifically, haplotypes I, II, and III were detected in 339, 40, and 28 accessions, respectively. The mean GV of haplotype II (789.88 ± 8.58) was significantly lower than that of haplotypes I (798.50 ± 8.29) and III (799.19 ± 4.60) (Fig. 3c, d). Moreover, one of the four significant SNPs (Excalibur_c11245_880) was detected at 3315 bp in the *TraesCS6B01G295400* coding sequence (CDS). The SNP (G/A) leads to changes in amino acids from arginine to histidine (Fig. 3e). The RNA-seq data revealed that this gene was highly expressed in developing seeds at 6 days post-anthesis (Fig. 3f).

## Discussion

GWAS have been conducted to analyze the yield and quality of various crops, including rice [21], soybean [22], cotton [23], and sorghum [24]. The size of the study population is closely related to the accuracy of the association analysis. Long et al. [25] reported that increasing the population size increases the number of individuals with rare alleles, thereby increasing the accuracy and efficiency of the positioning of rare alleles. Therefore, our GWAS for yield- and quality-related traits involved a wheat 90 K SNP array for 543 wheat accessions in multiple environments.

We completed a broad-scale comparison of the results of our study and those of previous investigations. For PH, a locus (RAC875_rep_c105718_585) on chromosome 4D identified in four environments based on BLUP data is about one LD from a QTL (QPH.caas-4DS) described by Li et al. [26], This previously identified QTL was also stably identified in two Chinese bread wheat populations. A QTL (QKNS.caas-4AL) for KNPS between markers Kukri_rep_c106490_583 and RAC875_c29282_566 on chromosome 4A was detected earlier in four environments by Gao et al. [13]. Two sites (Excalibur_c9370_966 and wsnp_Ra_rep_c70233_67968353) related to KNPS revealed in our study are located within this QTL. Gao et al. also reported a stable QTL (QTKW.caas-4BS.1) for TGW between markers BobWhite_c162_145 and Kukri_c66885_230 on chromosome 4B. In the present investigation, a predicted TGW-associated SNP locus (Tdurum_contig97386_207) was detected between two markers on chromosome 4B. Additionally, *TaAPO-A1/WAPO-A1* (*TraesCS7A02G481600*) was identified as a candidate gene for SNS through map-based cloning [27–29]. Two loci related to SNS (BS00026622_51 and RAC875_c19111_628) on chromosome 7A identified in four environments based on BLUP data are within one LD of *TaAPO-A1*. Dobrovolskaya et al. [30] cloned a *WFZP* (*WHEAT FRIZZY PANICLE*) gene related to SNS on chromosome 2D. This gene is within one LD of a stable SNS-related locus (GENE-0787_85) revealed in the current study. For the quality-related traits, our GPC locus (GENE-0411_807) overlaps the GPC QTL mapped to chromosome 1A by Kumar et al. [31]. Li et al. [32] identified a locus (IWB41869) related to starch granules on chromosome 7B, which is close to the FC locus (RAC875_c26057_370) we detected on the same chromosome.

In addition, we have also identified new stable SNPs that affect specific genes and have been detected in multiple environments. For example, *TraesCS7A01G482000*, which is related to SNS, was predicted to encode a haloacid dehalogenase (HAD)-like hydrolase domain-containing protein. In rice, the overexpression of *OsHAD1* reportedly leads to enhanced phosphatase activity and increased total and soluble P contents in Pi-deficient transgenic seedlings during the early panicle development stage. Increasing the P uptake rate can promote spikelet formation [33, 34]. The RNA-seq data available in an online wheat gene expression database indicated this gene is highly expressed when two nodes are detectable, which coincides with a key period for wheat spikelet development.

Lodging resistance is another important yield-related trait. Four significant SNPs for internode diameter were identified on chromosome 4B. These SNPs were detected within *TraesCS4B01G343700*. In Arabidopsis, *AtVPS25* (vacuolar protein sorting-associated protein) regulates auxin biosynthesis via its effects on the expression of specific auxin-related genes. An increase in the auxin content of wheat plants may lead to increased stalk diameters and enhanced lodging resistance [35–37]. A transcriptome-level analysis of *TraesCS4B01G343700* in different tissues and developmental stages proved that this gene is highly expressed during the stem and internode development stage. The expression level peaked in the milk grain stage, implying the changes occurring in this stage have important implications for the lodging resistance of wheat plants.

Regarding the quality-related trait GV, we identified *TraesCS6B01G295400*, which encodes a LisH domain-like protein, as a candidate gene. In rice, *OsLIS-L1* (lissencephaly type-1-like 1 protein) is important for male gametophyte formation, with mutations to this gene resulting in abnormal development. Additionally, the protein encoded by this gene influences grain characteristics and is closely related to the floral development and grain-filling stages [38]. An analysis of publicly available wheat RNA-seq data revealed that this gene is highly expressed in developing seeds, specifically in the starchy endosperm from day 6 to day 14 post-anthesis and showed a gradually decreasing trend. Accordingly, this gene is important for the early grain-filling stage.

## Conclusions

In summary, we conducted a GWAS based on the wheat 90 K SNP array to investigate the yield- and quality-related traits in 543 major wheat accessions. The resulting data were analyzed to identify relevant SNP loci and candidate genes. We are currently developing Kompetitive Allele-Specific PCR markers for the significant loci. These markers will enable researchers and breeders to efficiently transfer alleles into elite wheat genotypes [39]. Additionally, a more thorough characterization of the candidate genes described herein may enhance our understanding of the molecular mechanisms regulating wheat yield and quality.

## Methods

### Plant materials and experimental design

A bread wheat panel of 543 genotypes including cultivars, regional test lines, and introduced parental lines was used, the details have been published in our previous paper [20]. During the two growing seasons, wheat plants grow in three places in Hebei Province. The locations were Baoding (115.5°48′E, 38°85′N), Cangzhou (116°80′E, 38°58′N), and Xingtai (118°9′E, 39°42′N). The six environments were designated as follows: 2016 Baoding (E1), 2016 Cangzhou (E2), 2016 Xingtai (E3), 2017 Baoding (E4), 2017 Cangzhou (E5), and 2017 Xingtai (E6). The field trial was completed using a completely randomized design. Each plot contained three 1.5 m rows with 0.25 m between rows. The plant spacing is about 2.5 cm. Wheat plants were cultivated following normal local practices.

### Phenotypic evaluation

Twenty-five phenotypic traits were measured, including growth and development-related traits (FLL, FLW, FLA, FA, MTN, HD, MP, GFP, GFR, TGW, PH, FD, FIL, SD, SIL, and TH), yield-related traits (SL, SNS, KNPS, PET, and EPM), and quality-related traits (GV, GPC, WGC, and FC). The data recorded for each trait are summarized in Table S1. The phenotypic traits were assessed in all six environments. The phenotypic data for each environment and the BLUP data were used for the genome-wide association analysis.

### Phenotypic data analysis

The descriptive statistical analysis and correlation analysis for the phenotypic data were completed using the SPSS 25.0 software. Pearson’s correlation coefficients were calculated to evaluate the correlations among the traits.

### SNP genotyping

The wheat 90 K Illumina Infinium SNP array was used to genotype the association panel containing 543 accessions. The SNP data were clustered and automatically called using the Illumina BeadStudio genotyping software (Illumina, San Diego, CA, USA). The data were filtered to remove alleles with a detection rate less than 0.1 and a minor allele frequency less than 0.05 [12]. Additionally, samples with a loss rate greater than 10% and a heterozygosity frequency greater than 20% were eliminated.

### Genome-wide association analysis

The population structure, relative kinship, and LD were analyzed in a previous study [20]. In the current study, we completed a GWAS using the GAPIT package [40] in the R software. A mixed linear model program (Q + K) [41], with the population stratification results and kinship as covariates, was used to minimize false positives [40]. The *P* value threshold was calculated based on the number of markers (*P* = 1/n, *n* = total number of SNPs used) as described by Li et al. [42]. Regarding the GWAS results, a *P* value of 1/11,140 (−log_10_*P* = 4.05) was used as the criterion for identifying significant SNPs.

### Prediction of candidate genes and expression analysis

The ‘Chinese Spring’ Genome database (IWGSC RefSeq v1.0, http: //www.wheatgenome.org/) was used for predicting candidate genes for the significant sites revealed by the genome-wide association analysis. Specifically, candidate genes around the significant sites were identified according to the differences in the LD decay distance among chromosomal groups. The expression profiles of putative candidate genes were analyzed using a wheat gene expression database available online (http://www.wheat-expression.com/). This database, which includes 850 wheat RNA-sequencing samples and an annotated genome, reveals the similarities and differences between homoeolog expression levels in diverse tissues, developmental stages, and cultivars [33, 43].

## Supplementary Information

**Additional file 1: Fig. S1** Manhattan plots of GWAS results (BLUP values) excluding SNS, FD and GV traits. The horizontal line represents the significance threshold (−log_10_*P* = 4.05).

**Additional file 2: Table S1** Categories and descriptive statistics for the 25 yield- and quality-related traits evaluated in six different environments. **Table S2** Correlations among 25 yield- and quality-related traits. **Table S3** Basic details regarding the SNP markers used for a genome-wide association study of 543 wheat genotypes. **Table S4** Information regarding the candidate genes identified based on BLUP data.

## Data Availability

All data generated or analyzed during this study are included in this article (and its supplementary information files) or are available from the corresponding author on reasonable request.

## Abbreviations

EPM: Spike number per mu
FA: Flag leaf angle
FC: Flour content
FD: Diameter of the first internode
FIL: Length of the first internode
FLA: Flag leaf area
FLL: Flag leaf length
FLW: Flag leaf width
GFP: Grain-filling period
GFR: Grain-filling rate
GPC: Grain protein content
GV: Grain volume
HD: Heading date
KNPS: Kernel number per spike
LD: Linkage disequilibrium
MP: Mature period
MTN: Maximum tiller number
PET: Percentage of spike-bearing tillers
PH: Plant height
QTL: Quantitative trait locus
SD: Diameter of the second internode
SIL: Length of the second internode
SL: Spike length
SNS: Spikelet number per spike
TGW: Thousand grain weight
TH: Thrust
WGC: Wet gluten content
