# Supplementary material for: Genome-wide association study reveals the genetic basis of yield- and quality-related traits in wheat
Source: BMC Plant Biol. 2021 Mar 19;21:144. doi: 10.1186/s12870-021-02925-7 (PMC7980635; doi:10.1186/s12870-021-02925-7)
Supplement: Supplementary file 1 — Additional file 1: Fig. S1 Manhattan plots of GWAS results (BLUP values) excluding SNS, FD and GV traits. The horizontal line represents the significance threshold (−log10P = 4.05). [file 12870_2021_2925_MOESM1_ESM.docx]

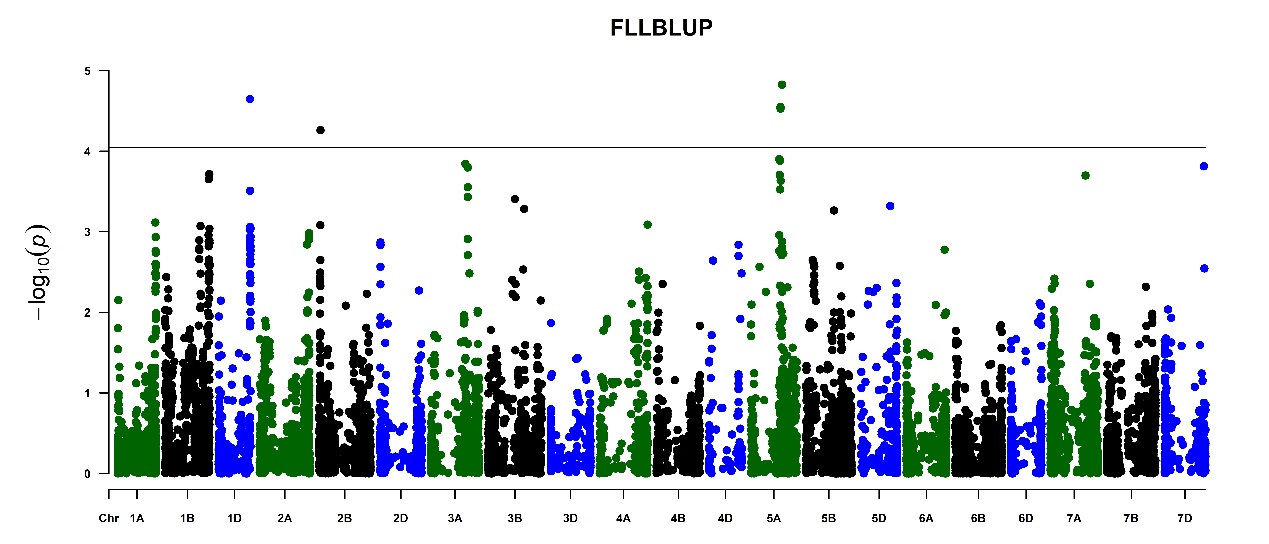


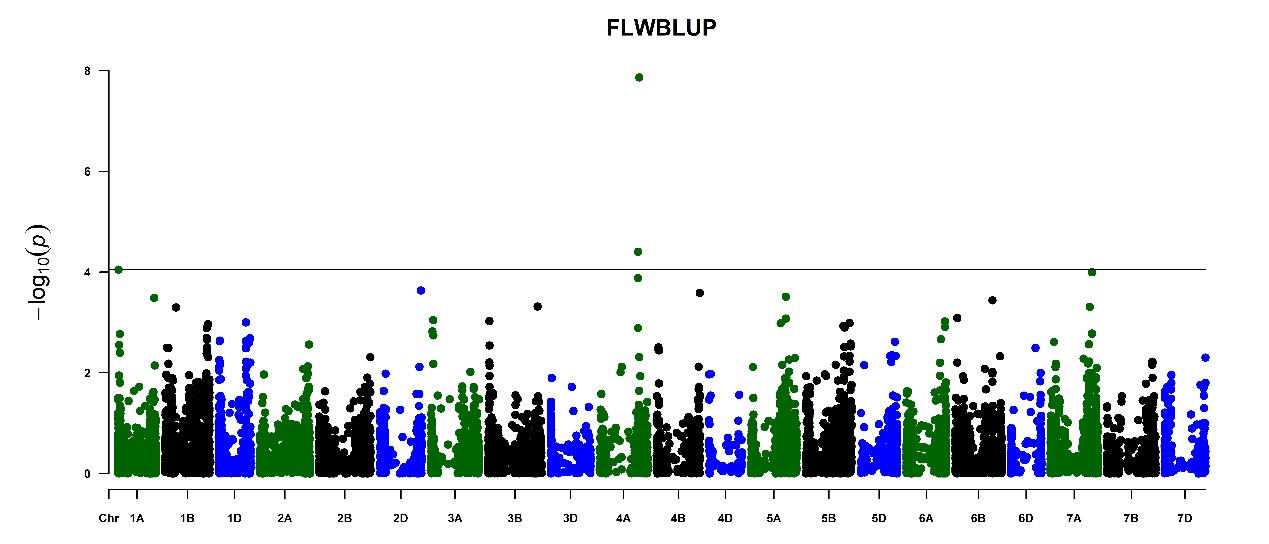


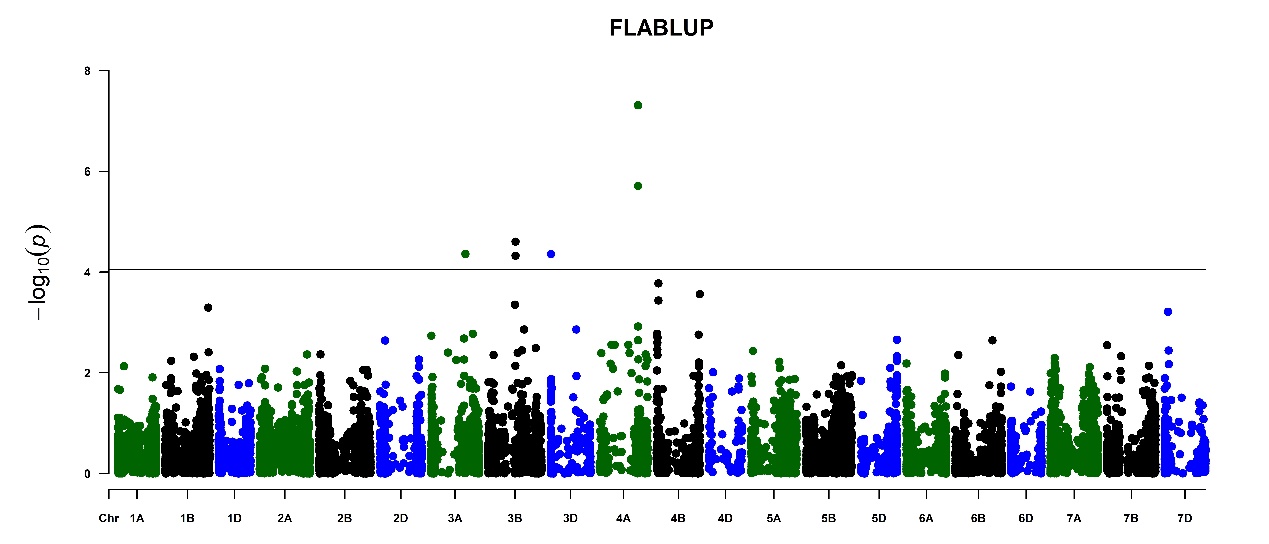


**
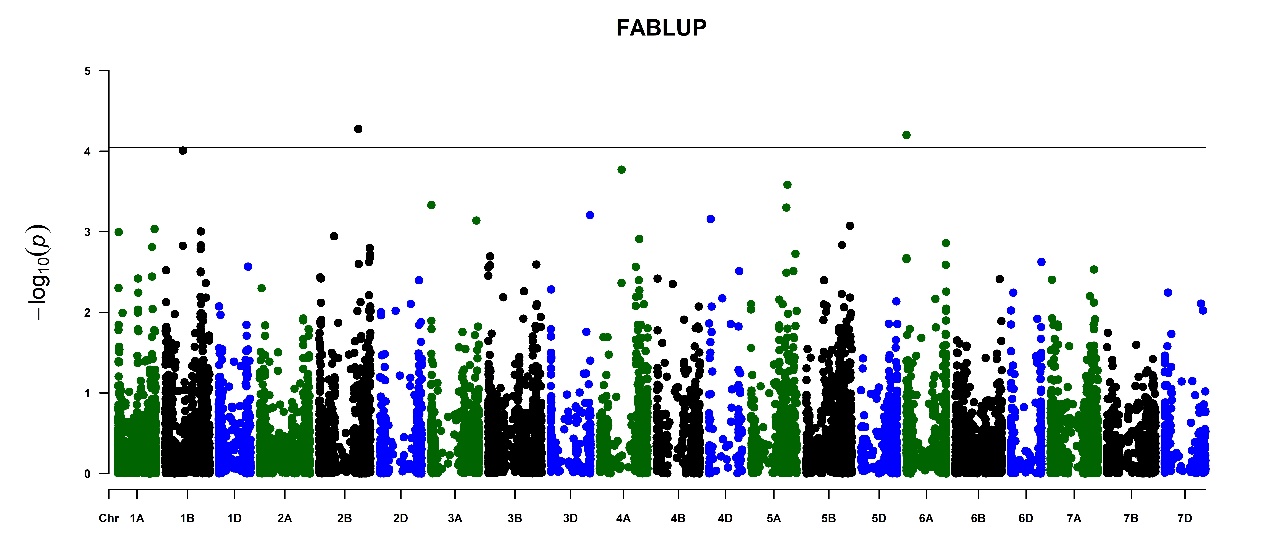
**


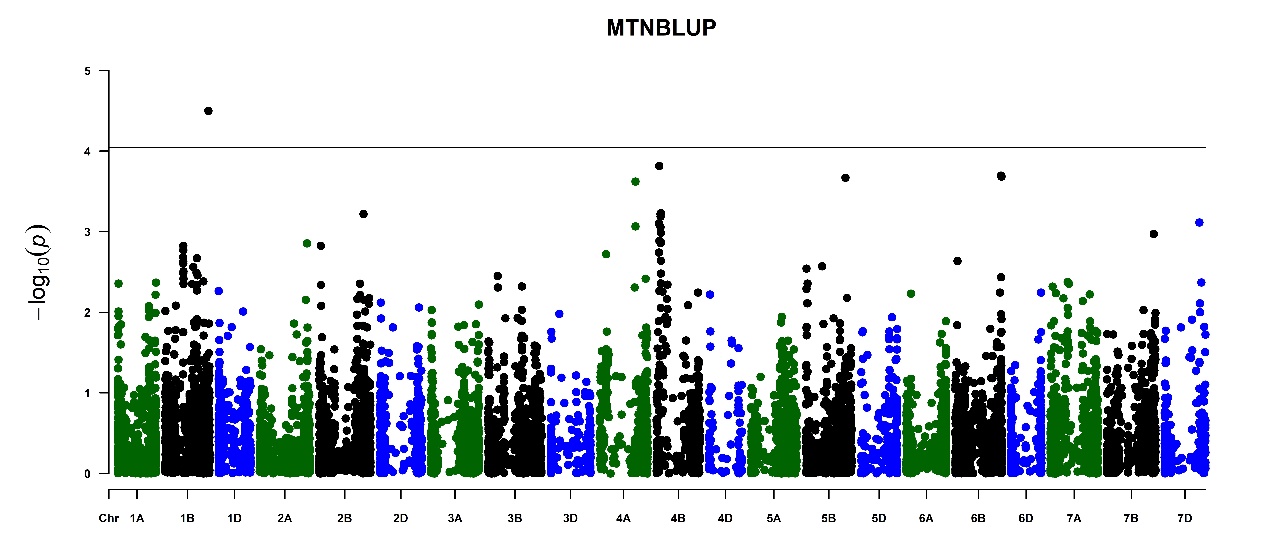


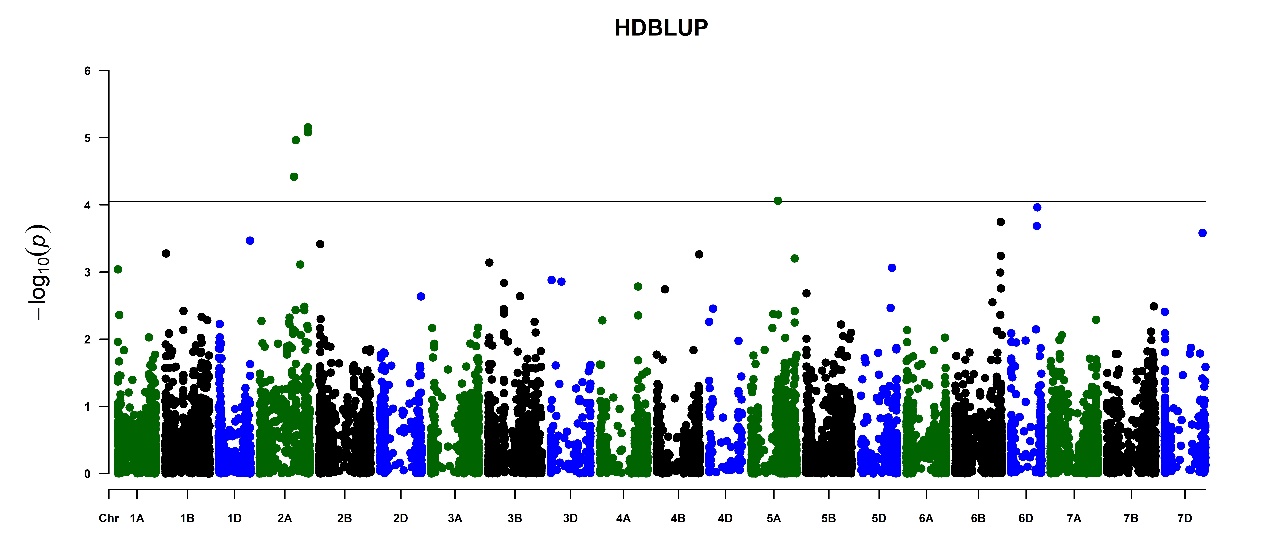


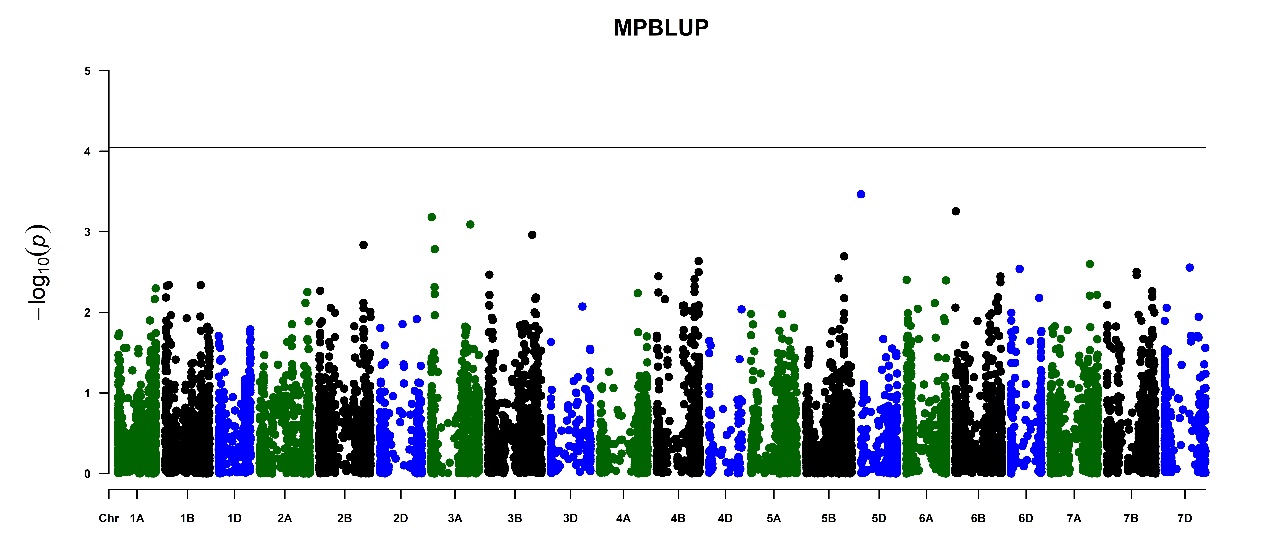


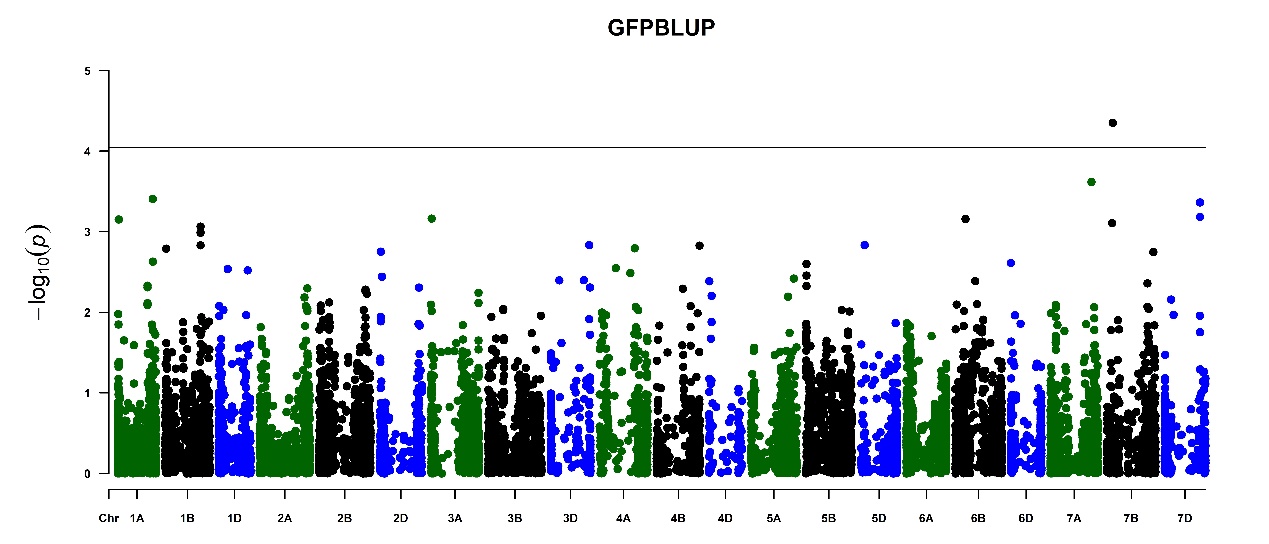


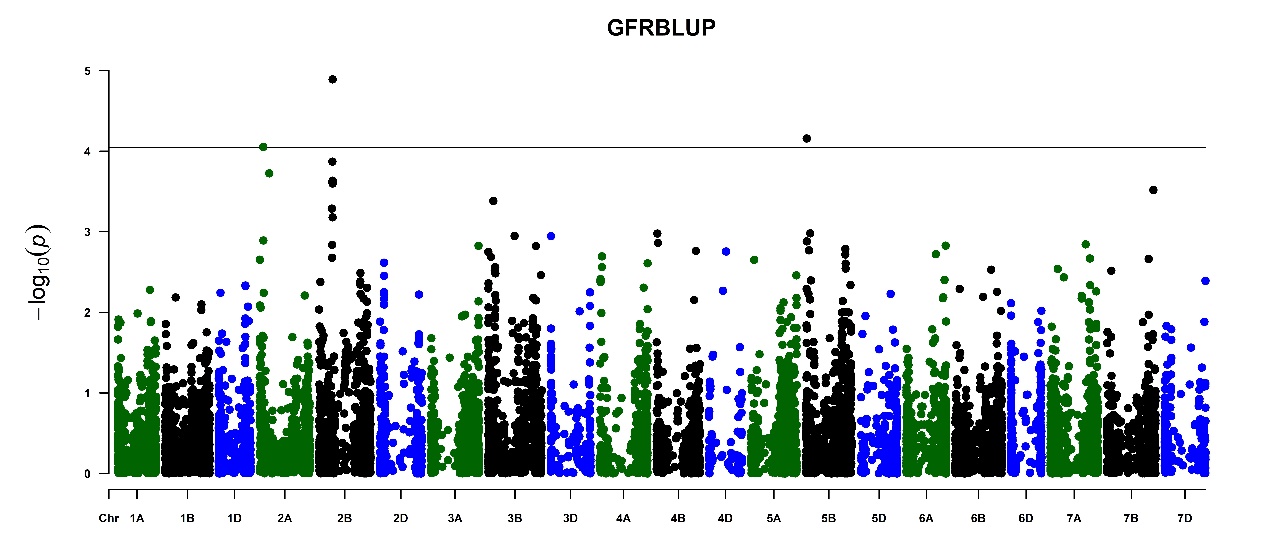


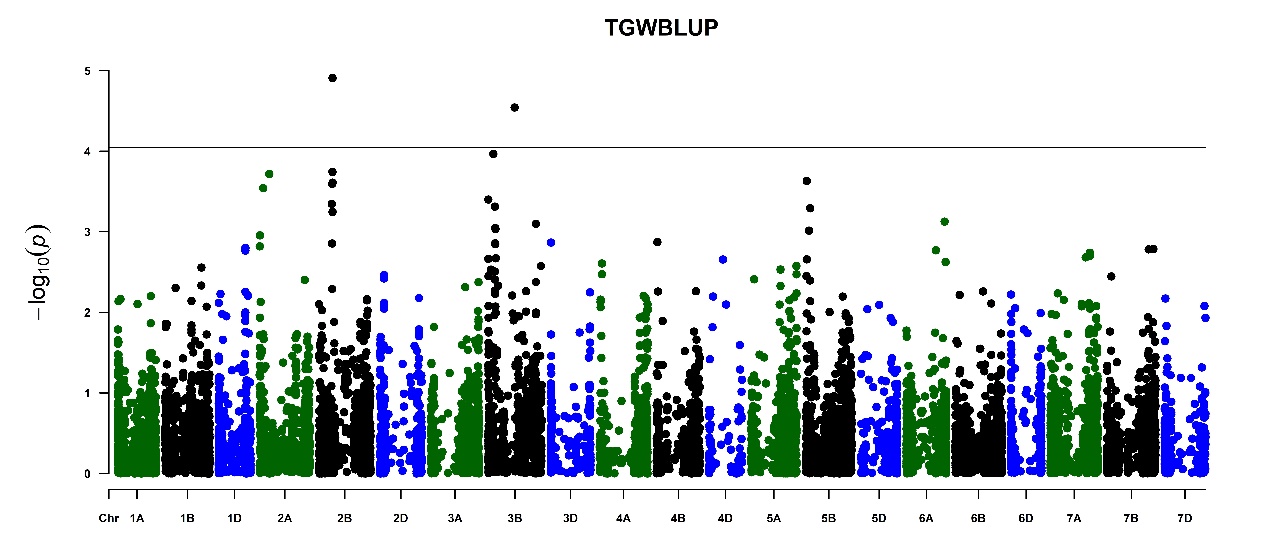


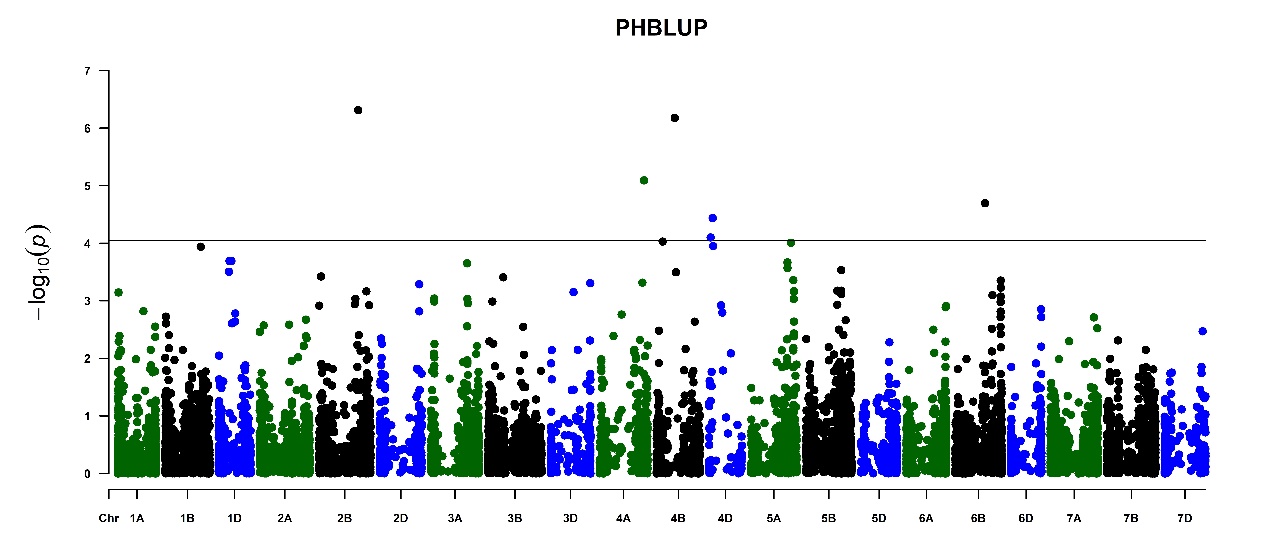


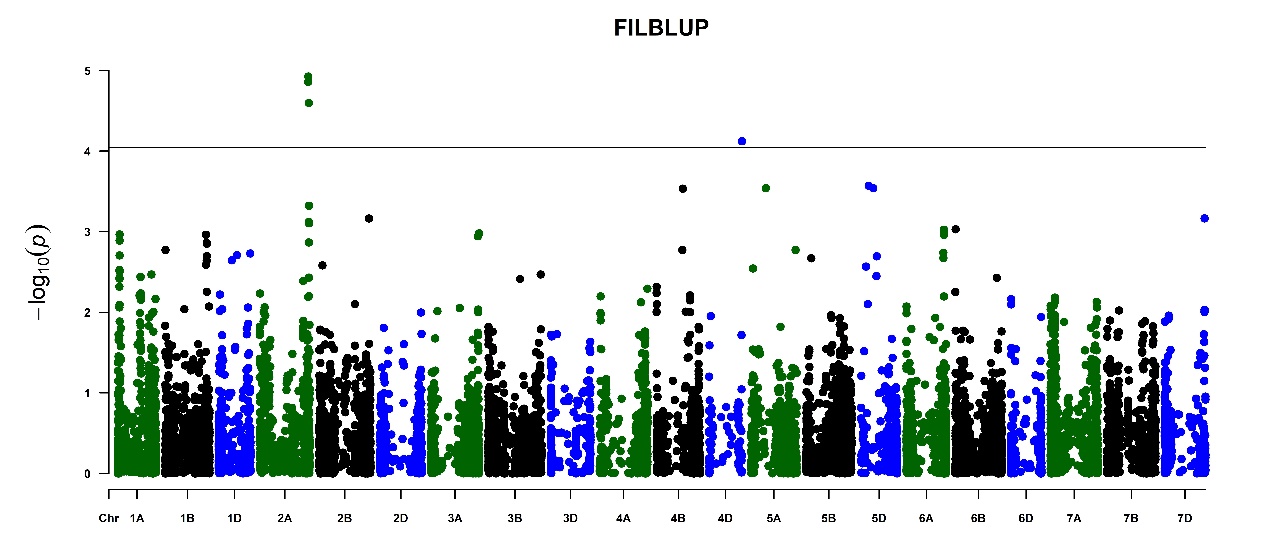


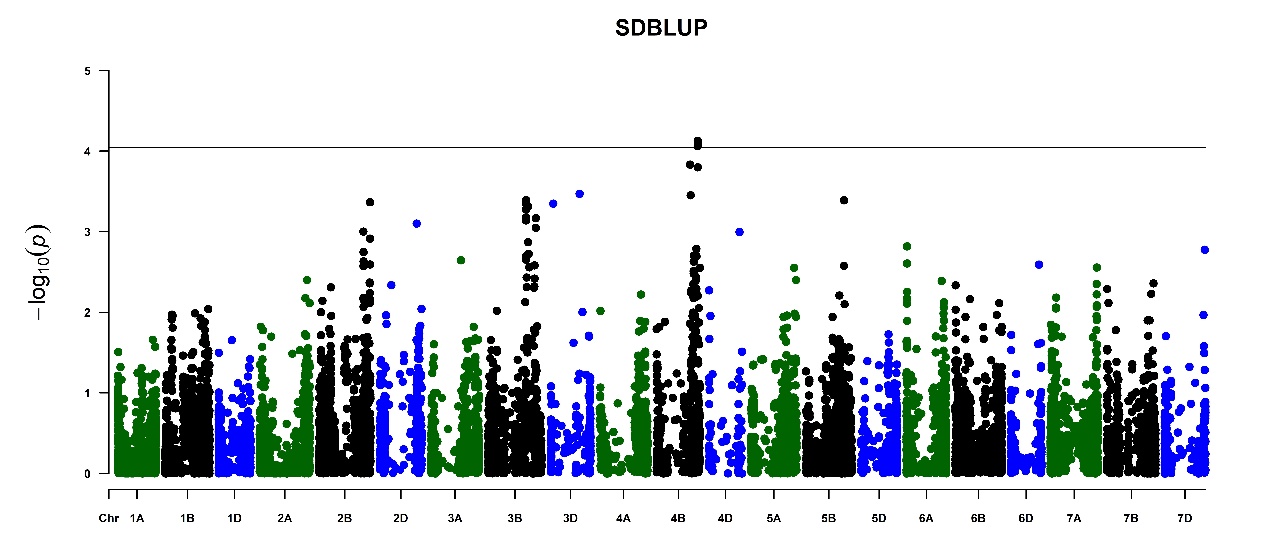


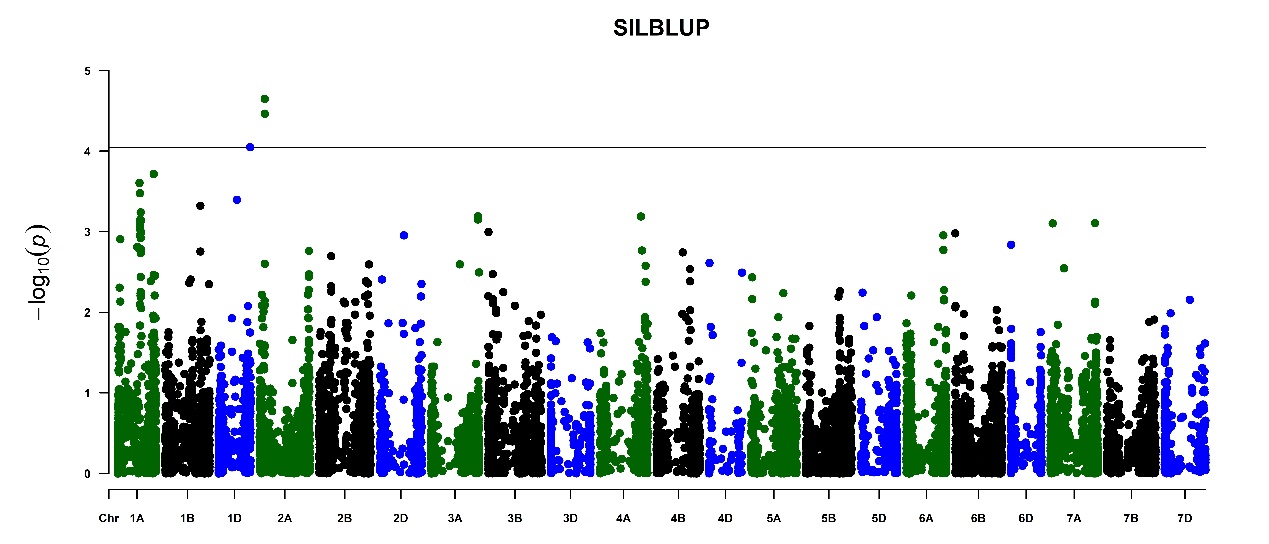


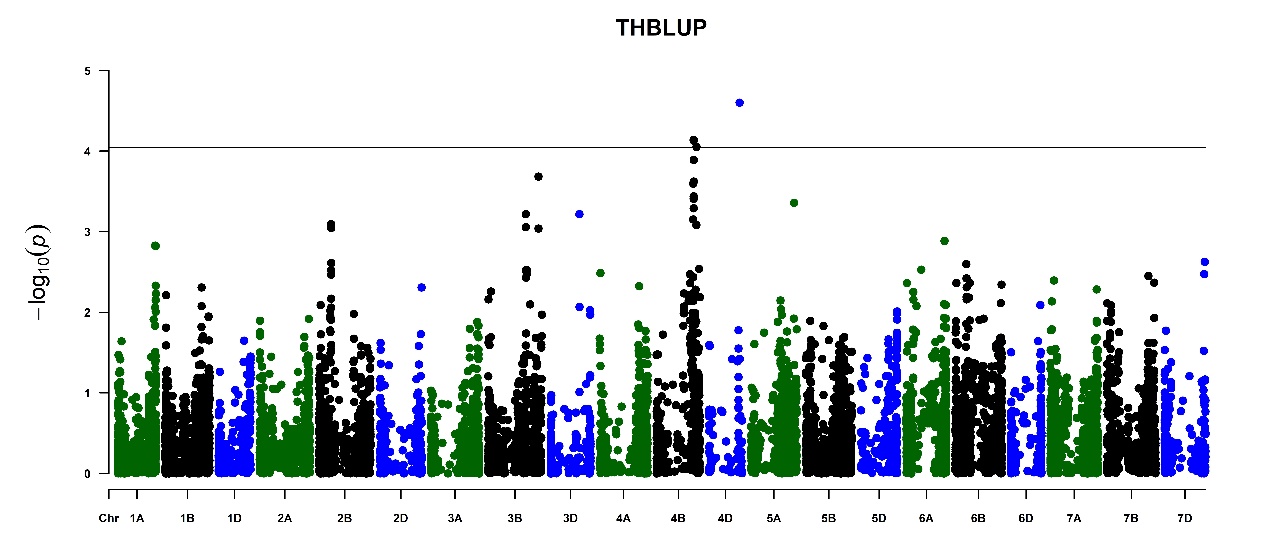


**
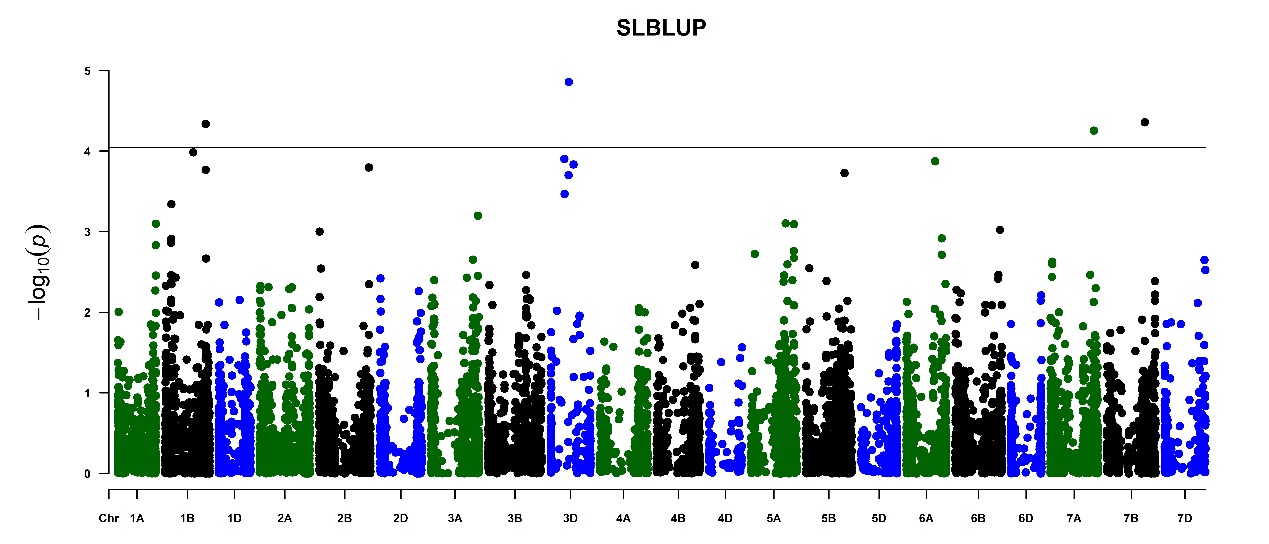
**


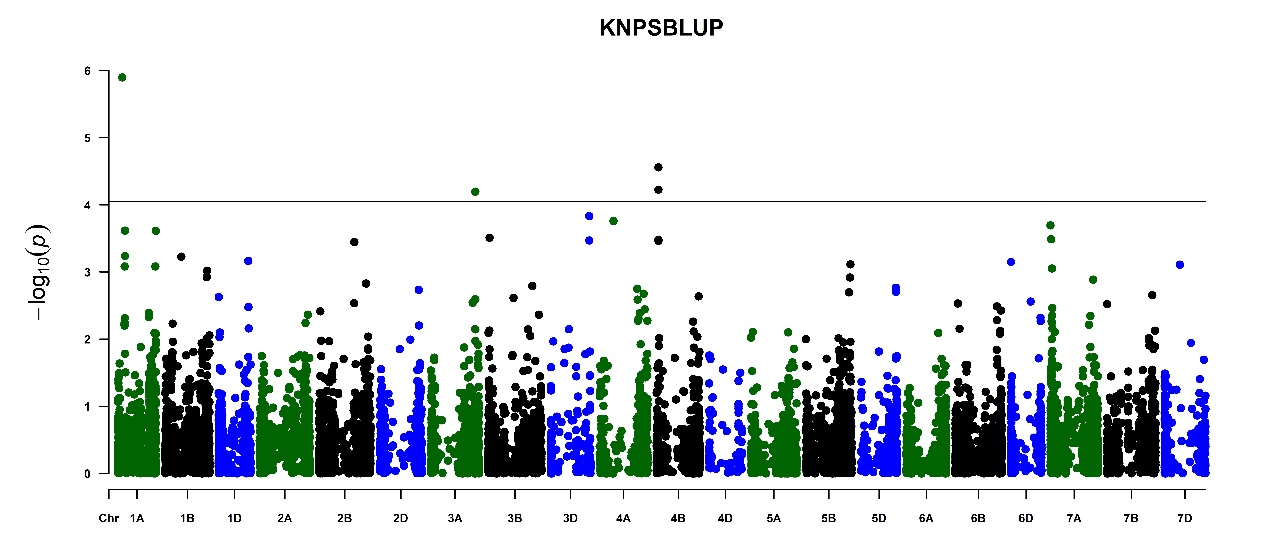


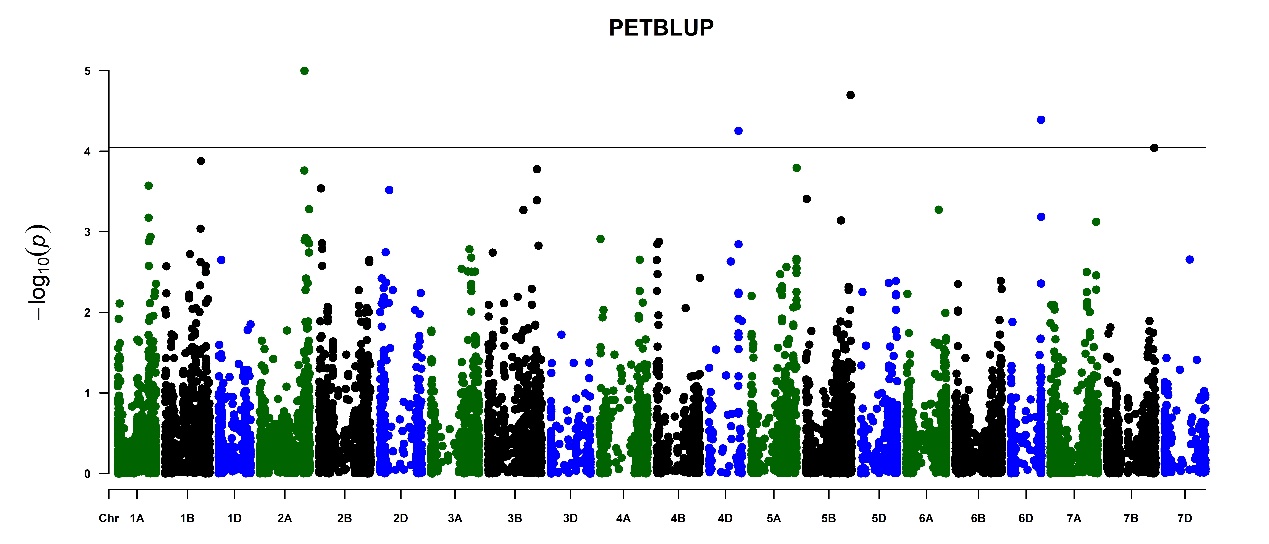


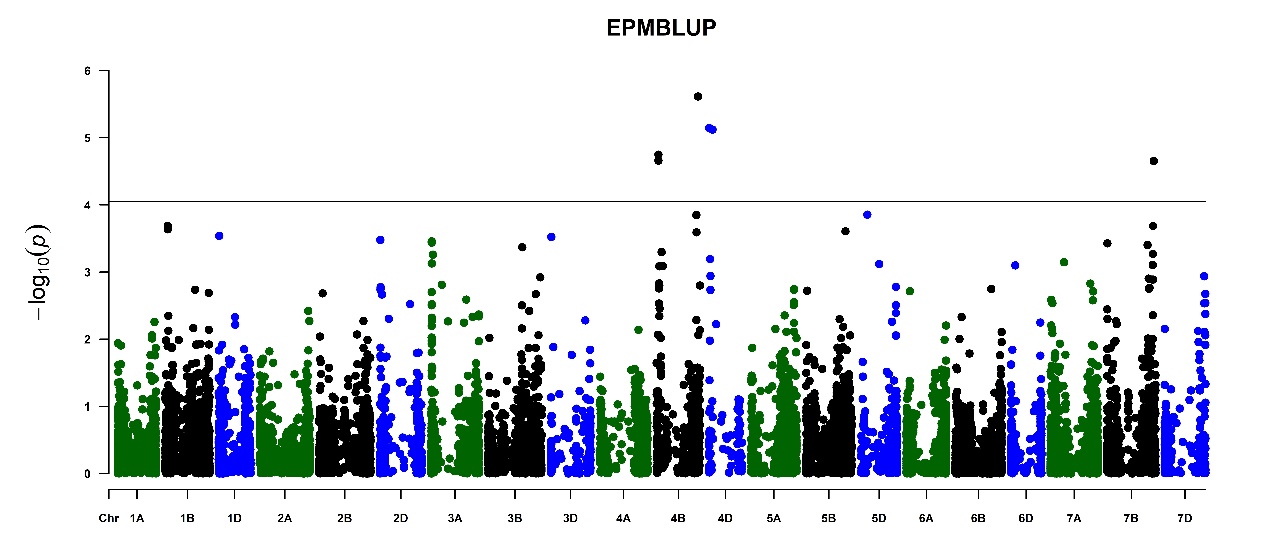


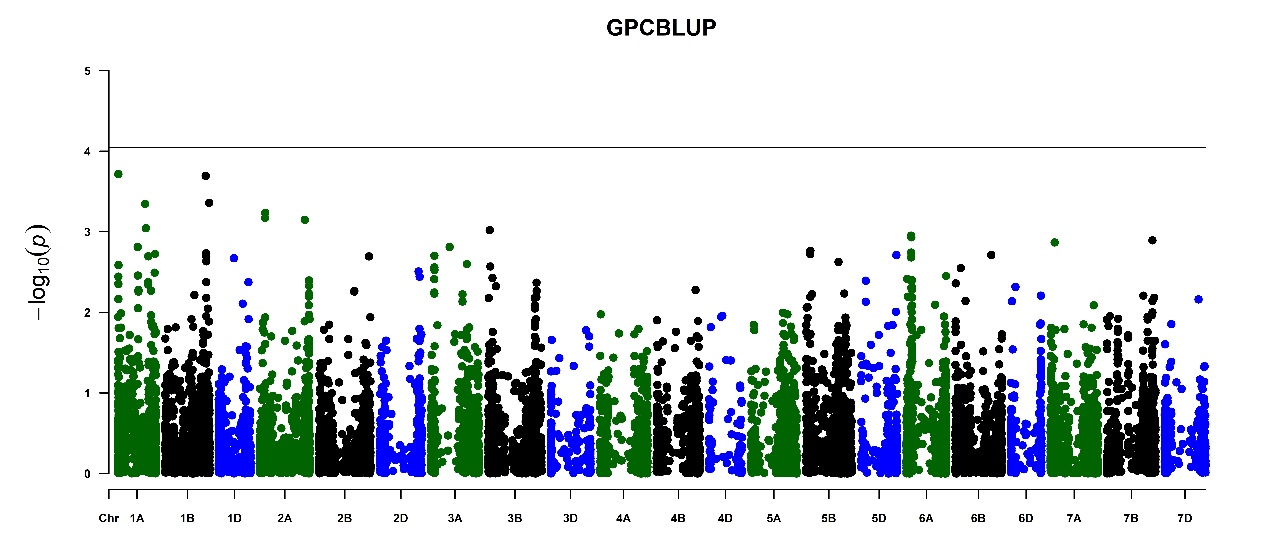


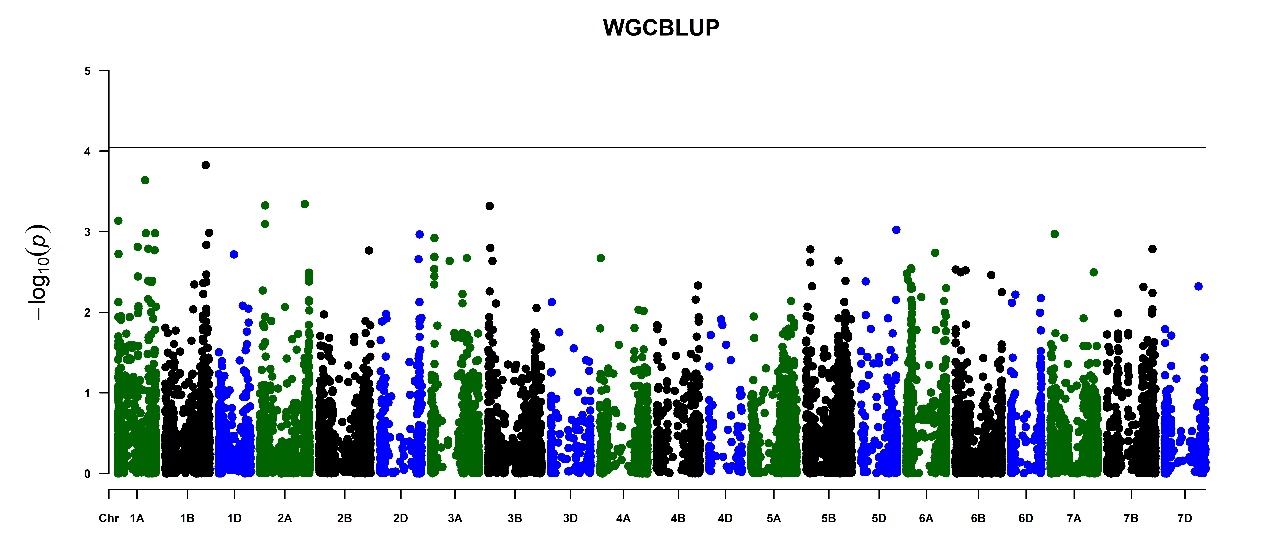


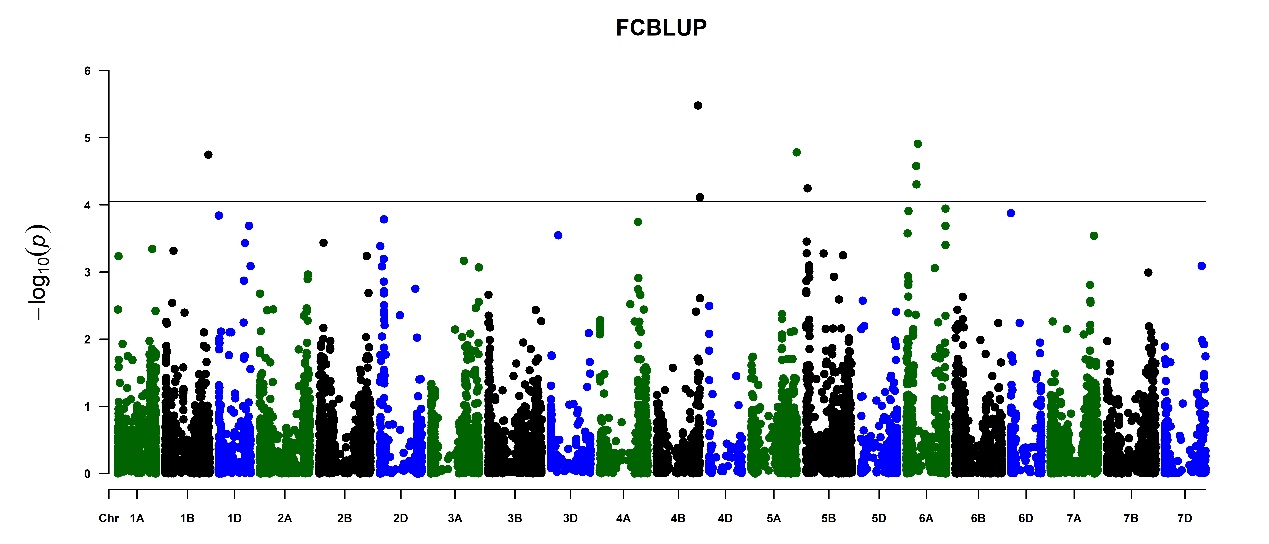


**Fig. S1** Manhattan plots of GWAS results (BLUP values) excluding SNS, FD and GV traits. The horizontal line represents the significance threshold (−log_10_*P* = 4.05).
